# Hydroxyl Group Separation Distances in Anti-Freeze Compounds and Their Effects on Ice Nucleation

**DOI:** 10.3390/ijms21228488

**Published:** 2020-11-11

**Authors:** Monika Bleszynski, Matt Reil, Maciej Kumosa

**Affiliations:** 1 Department of Mechanical & Materials Engineering, Ritchie School of Engineering and Computer Science, University of Denver, Denver, CO 80208, USA; matt.reil@du.edu (M.R.); mkumosa@du.edu (M.K.); 2 NSF I/UCRC for Novel High Voltage/Temperature Materials and Structures, University of Denver, Denver, CO 80208, USA

**Keywords:** ice nucleation, molecular dynamics, polyvinyl alcohol, hydroxyl distance, AFGP

## Abstract

Since the discovery of biological antifreeze glycoproteins (AFGPs), which can inhibit ice nucleation, there has been considerable interest in understanding their mechanisms and mimicking them in synthetic polymers. In this study, we used molecular dynamics simulations of modified polyvinyl alcohol (PVA) compounds to show that the hydroxyl (OH) group distance is a key factor in whether certain compounds promote or inhibit ice nucleation. A hydroxyl distance smaller than ~2.8 Å but greater than ~7.1 Å in modified PVA (MPVA) compounds was associated with the promotion of ice nucleation, while a hydroxyl group separation distance of approximately ~5.0 Å was correlated with a delay in ice nucleation, owing to changes in the energy of the system. Thus, these results may help explain some of the mechanisms of current known anti-freeze compounds and may have implications for designing new anti-freeze compounds in the future.

## 1. Introduction

Various methods have been presented for preventing ice formation, including low ice adhesion surfaces and anti-freeze agents [1,2,3,4,5]. In order to develop new anti-freeze agents, many have sought to mimic biological antifreeze proteins (AFPs), which play critical roles in preventing ice formation in some plants, fish, and insects indigenous to cold climates [6,7,8,9,10,11]. AFPs are polypeptides that are able to interact with ice crystals, controlling and limiting the growth and formation of ice that may be damaging to an organism [6,7,8].

In order to survive in sub-freezing waters, animals such as the Antarctic Notothenioids (*Boreogadus saida*) produce specific types of AFPs known as antifreeze glycoproteins (AFGPs), which limit the formation of ice in their veins, allowing them to inhabit waters ranging from 0 °C to −4 °C [9,10,11,12,13,14]. Studies have shown that AFGPs and AFPs can bind to different ice faces depending on the glycoprotein type, thereby inhibiting the growth of the ice crystal [9,10,11,12,13,14]. While some AFPs rely on methyl groups for ice binding via anchored clathrates [6,7,8], certain AFGPs utilize hydroxyl groups for their functionality [10,11,12,13,14].

**Figure 1:**
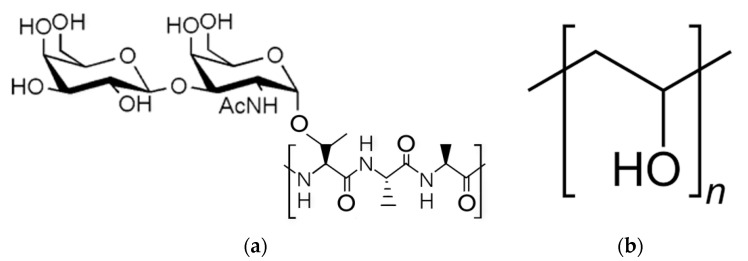
Structure of native antifreeze glycoprotein (AFGP-8) (**a**) and polyvinyl alcohol (PVA) monomer (**b**).

Unfortunately, the instability and high cost of AFGPs make them impractical for large-scale commercial applications [10,11,12,13,14]. Understanding and recreating the non-colligative freezing point depression and ice recrystallization inhibition (IRI) properties of AFGPs have therefore been of particular interest in areas of science, consumer products, and medicine [11,12]. Polyvinyl alcohol (PVA) has been identified by several studies as a compound that can inhibit ice nucleation in solution [15,16,17,18,19,20,21,22,23,24,25,26]. Although it lacks the complex polypeptide backbone of most AFGPs, PVA is similar to AFGPs in that it contains primarily hydroxyl (OH) groups, which have been found to be a key element in the IRI effect of PVA (Figure 1a,b) [15,16,17,18,19,20,21,22,23,24].

PVA is formed through the hydrolysis of polyvinyl acetate and can be fully or partially hydrolyzed [18,19,20,21,22,23]. When fully hydrolyzed, PVA exhibits strong inter-molecular hydrogen bond interactions due to the close proximity of the OH groups, while partially hydrolyzed PVA does not due to the intermediate placement of COCH3 groups [15,16,17,18,19,20,21,22,23]. While some molecular-level specifics of ice crystallization remain unclear, recent research has suggested a correlation between the IRI of antifreeze proteins and lattice matching with the ice structure [24]. Although many studies have investigated the effects of PVA on ice nucleation, there is an inconsistency with regard to the ice nucleation activity of PVA, as some studies have shown that PVA can either promote or inhibit ice nucleation [25,26,27,28]. For example, Mochizuki, et al. demonstrated that PVA can promote homogeneous ice crystallization by destabilizing the liquid phase, while work done by Budke et al. determined the IRI activity of partially hydrolyzed PVA can be attributed to the Gibbs–Thomson effect [21]. This difference is especially noteworthy, considering that the differences in partially and fully hydrolyzed PVA lie in the substitution of the hydroxyl (OH) functional groups along the PVA backbone [18,19,20,21,22,23].

Weng et al. recently provided direct evidence of a geometrical match between PVA and ice, where the oxygen atoms from PVA hydroxyl groups will form hydrogen bonds with the ice structure, assuming that the O…O distance is not greater than 3.5 Å and the O-H-O bond angle is greater than 145° [23]. Hydroxyl oxygen atoms geometrically matched the ice lattices by replacing the oxygen ice atoms, where the radial distribution function between the O_ice_–O_ice_ has a first primary peak at 2.75 Å and a secondary peak at 4.5 Å [23].

**Figure 2:**
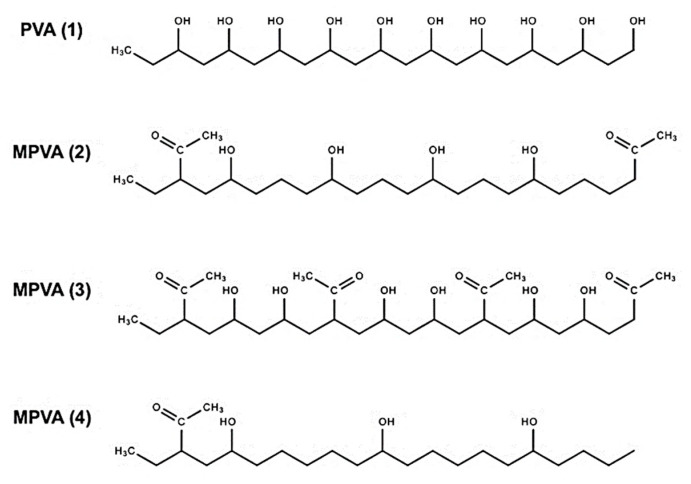
Structures of polyvinyl alcohol molecules: PVA (**1**), MPVA (**2**), MPVA (**3**), and MPVA (**4**) used in the MD ice nucleation simulations in this study.

Given this evidence, it can be presumed that the effects of PVA on ice nucleation are critically dependent on the polymer’s hydroxyl separation distance and quantity, in order for a geometrical match to occur. Therefore, while other studies have addressed the effects of partially or fully hydrolyzed PVA and PVA length, we sought to address the effects of hydroxyl separation distance on ice nucleation by observing four molecules: a standard PVA and three modified PVA (MPVA) 10 mer molecules that were constructed from a base PVA structure. Each compound was created with varying distances between the hydroxyl (OH) groups, as shown in Figure 2, to observe how the substitution and modification of hydroxyl groups could seemingly cause a compound such as PVA to swing from promoting to inhibiting ice nucleation [15,16,17,18,19,20,21,22,23,24,25,26,27,28]. In this study, MPVA compounds were modified from standard PVA, and were constructed by eliminating or modifying hydroxyl groups while still retaining the original backbone.

## 2. Results and Discussion

**Figure 3:**
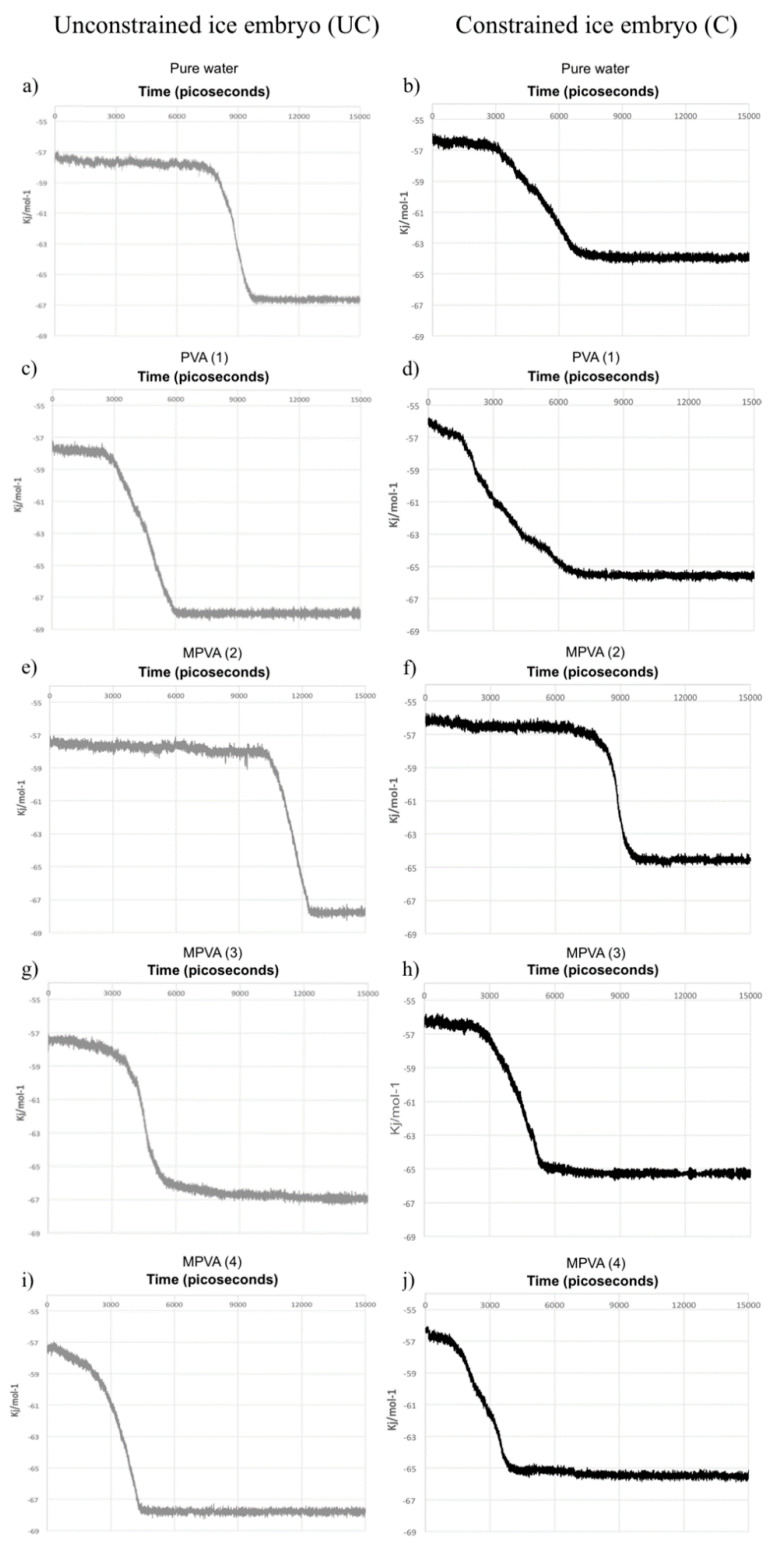
Progression of ice nucleation in water droplets with unconstrained (UC) and constrained (C) ice embryo as a function of total potential energy for pure water (**a**,**b**), and water with: PVA (1) (**c**,**d**), MPVA (2) (**e**,**f**), MPVA (3) (**g**,**h**), and MPVA (4) (**i**,**j**).

**Figure 4:**
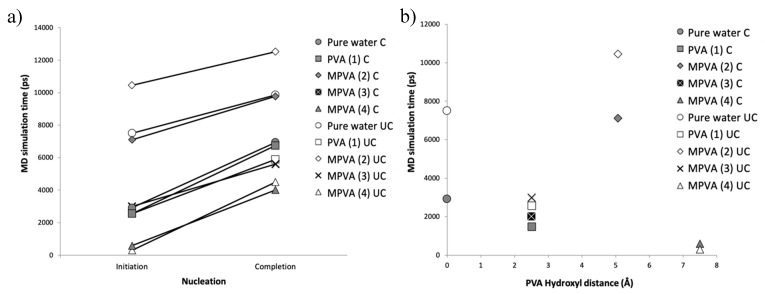
Initiation and completion of water droplet nucleation for constrained (C) and unconstrained (UC) ice embryo (**a**), and MD nucleation initiation times as a function of initial PVA and MPVA hydroxyl distances (**b**).

The nucleation results, as a function of time (ps), are shown in Figure 3a–j. Nucleation was assumed to occur when the total potential energy of each system abruptly decreased. All simulations with one molecule of PVA (*n* = 10) resulted in the nucleation of hexagonal ice, with notable differences. MD nucleation simulations showed a clear promotion of ice nucleation in the presence of PVA (1), MPVA (3), and MPVA (4) compared to the nucleation of pure water, regardless of ice embryo constraint (Figure 3a–j). Ice nucleation initiated the fastest in the presence of MPVA (4), with nucleation beginning almost immediately at 300 ps. This was followed closely by PVA (1) and MPVA (3), which both initiated nucleation sooner than pure water. Nucleation of pure water initiated at 7510 ps and 2970 ps for unconstrained and constrained simulations, respectively (Figure 3a,b and Figure 4a,b).

By contrast, MPVA (2), which had an average hydroxyl distance of ~5 Å, extended the initiation of nucleation compared to pure water by a significant margin (Figure 3e,f). Nucleation was initiated at approximately 10,450 ps and 7100 ps for unconstrained and constrained nuclei, respectively (Figure 4a,b). Nucleation was therefore delayed by approximately 40% and 144%, for unconstrained and constrained ice embryo simulations, respectively.

The average hydroxyl distances in all PVA molecules changed after nucleation was complete, with MPVA (2) exhibiting an increase of 0.68 Å, and MPVA (4) decreasing by 0.63 Å. The average hydroxyl distances after and before nucleation are shown in Table 1.

When measured as a function of minimum hydroxyl distance, only MPVA (2) with a final average hydroxyl distance of 5725 Å (Table 1) was correlated with a delay in ice. PVA and MPVA molecules with either smaller or larger initial hydroxyl distances promoted ice nucleation by shortening the time to nucleation compared to pure water, regardless of the presence of a constrained embryo (Figure 4).

**Figure 5:**
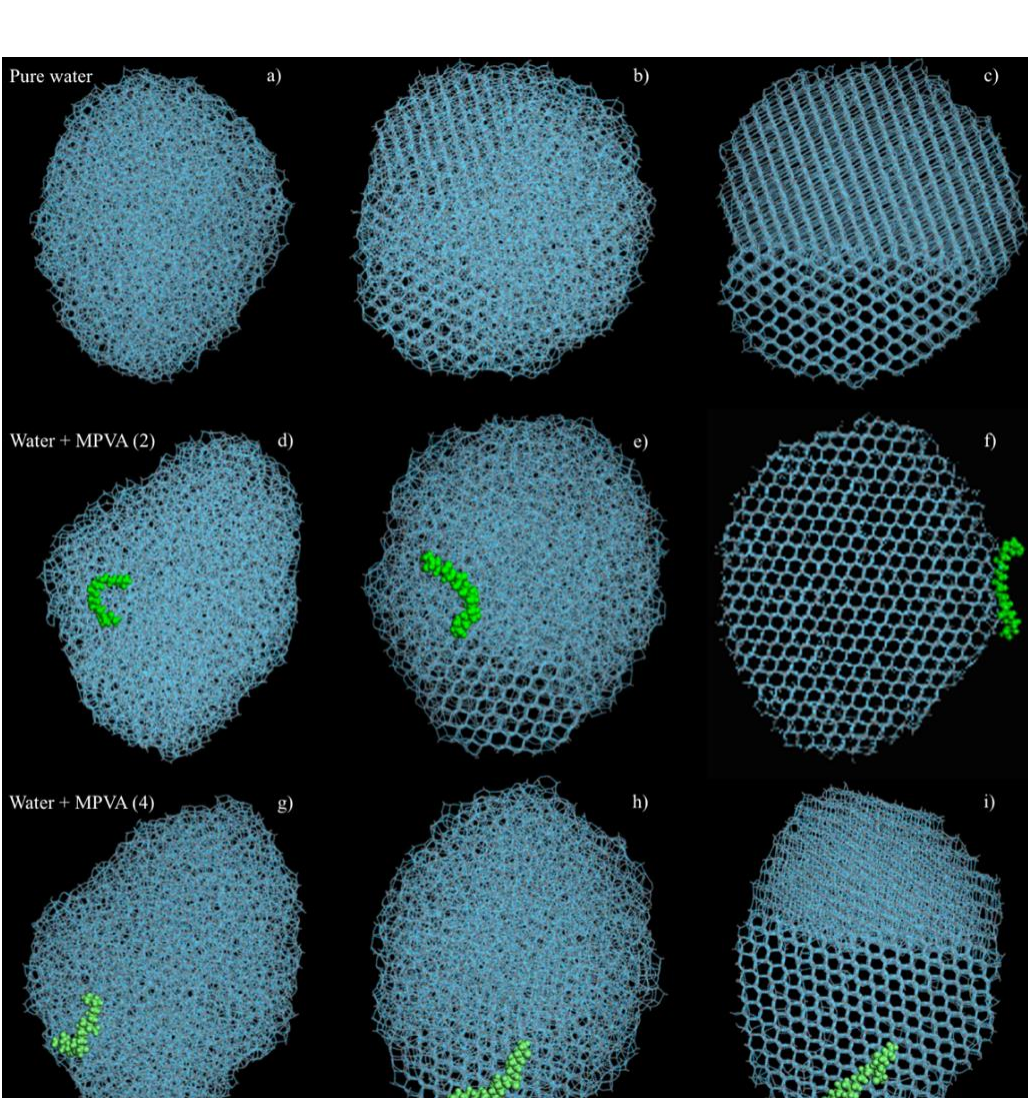
Progression of ice nucleation without a constrained ice embryo: (**a**–**c**) pure water, and water in the presence of MPVA (2), (**d**–**f**) and water in the presence of MPVA (4) (**g**–**i**).

The geometries of the final crystalline ice structures were also notably different for pure water and water in the presence of PVA and MPVA molecules (Figure 5a–i). In both pure water simulations (with and without constrained ice embryos), ice nucleation initiated at three separate locations and continued until the crystalline faces joined (Figure 5c), and the final nucleated ice structure consisted of two separate crystalline assemblies. Crystallization of the pure water droplet did not extend from the constrained ice embryo, but instead nucleated preferentially from the edge of the water droplet and continued through the water droplet in the *x*-axis, in both the constrained and unconstrained simulations. In simulations with PVA and modified PVA molecules, PVA (1), MPVA (3), and MPVA (4) molecules adsorbed on the 001 basal plane of the ice crystal after ice crystallization. By contrast, MPVA (2) adsorbed on the 100 plane (Figure 5d). Additionally, unlike MPVA (2), which moved significantly around the water droplet before crystallization (Figure 4d–f), PVA (1), MPVA (3), and MPVA (4) did not considerably migrate across the water droplet surface during or after nucleation (Figure 5g–i).

**Figure 6:**
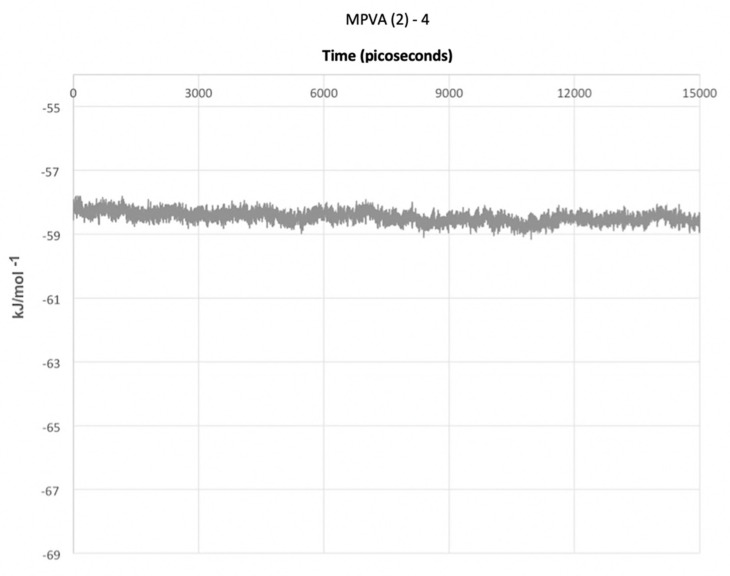
Total potential energy of the MD system containing a water droplet in the presence of four MPVA (2) molecules over 15,000 ps.

The final simulation of a water droplet with an unconstrained ice embryo and four MPVA (2) molecules produced entirely different results (Figure 6). The water droplet did not nucleate in the allotted time frame of 15,000 ps. This is in contrast to the results shown in Figure 3a–j which utilized single PVA and MPVA molecules, and which all resulted in the nucleation of the water droplet in less than 15,000 ps. The potential energy of the system with four MPVA (2) molecules did fluctuate and remained slightly lower than that of the system with a single MPVA (2) molecule.

**Figure 7:**
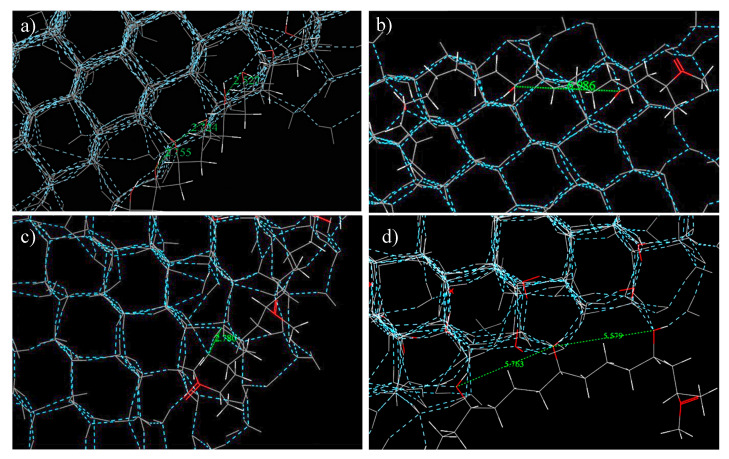
Hexagonal ice structures after nucleation (without constraints) in the presence of (**a**) PVA (1), (**b**) MPVA (4), and (**c**) MPVA (3), and (**d**) MPVA (2) with pentagonal ice structures.

Calculating the O_ice_–O_ice_ distances between the water molecules in the nucleated pure ice with no PVA model revealed an average of 2.689 Å, which is very close to initial/final hydroxyl distances of PVA (1) and MPVA (3): (2547/2828 Å and 2667/2855 Å, respectively). The presence of carbonyl groups in MPVA (3) appeared to only minimally affect the initiation of nucleation. Additionally, upon nucleation, PVA (1), MPVA (3), and MPVA (4) did not significantly disturb the hexagonal crystalline structure of the ice (Figure 6a–c). By contrast, MPVA (2) distorted the hexagonal structure where the hydroxyl groups were in contact with the water molecules (Figure 6d), forming pentagonal instead of hexagonal rings and stretching the hexagonal ice structure. This suggests that the distortion and stretching of the crystalline structure when in contact with MPVA (2) may be linked to the longer nucleation time of water when in the presence of MPVA (2), as the PVA molecule impedes normal hexagonal crystal formation. Therefore, it appears PVA (1), MPVA (3), and MPVA (4) could promote ice nucleation by either of two mechanisms: by reducing the total potential energy of the system or by acting as a scaffold for ice nucleation and growth (Figure 2a–j and Figure 7a–d).

The MD simulations showed drastic differences in nucleation activity depending on the hydroxyl distance along the polymer backbone among the various PVA and MPVA structures in this study. An increase or decrease in the distance beyond a critical value was shown to promote or accelerate ice nucleation. It can therefore be inferred that an ideal hydroxyl distance is required to delay or inhibit ice nucleation. For nucleation inhibition (*N_I_*) to occur, it appears that the hydroxyl distance (HD) for anti-freeze molecules must therefore be approximately greater than 2858 Å but less than 7117 Å: 2858Å < *N_I_* HD < 7117 Å.

Indeed, the small differences in hydroxyl distance and their effects on ice nucleation could help to explain the IRI activity of other well-known anti-freeze compounds, such as ethylene glycol and propylene glycol. The hydroxyl distances in ethylene glycol and propylene glycol, when measured using MD, are approximately 3832 Å and 2911 Å, respectively, when measured from O⋯O. Furthermore, the hydroxyl distances in AFGP-8 ranged between ~3455 Å to ~5518 Å, when measured using MD software. This distance can be correlated to the unit cell dimensions of hexagonal crystal ice, which has a lateral lattice spacing of 4518 Å, and 7356 Å (14–20), and in our study the average O_ice_⋯O_ice_ measured distances were 2.76 Å. Thus, the measured O_ice_⋯O_ice_ distances were very close to the hydroxyl distances of PVA (1) and MPVA (3) in this study.

It can be speculated that the similarity of the O_ice_⋯O_ice_ distance supports the notion that additives such as PVA can promote homogeneous crystallization by destabilizing the liquid phase when an ideal set of structures is present. Furthermore, the hydroxyl distance in MPVA (2) mimics that of liquid water, and the bending and twisting motion of the MPVA (2) polymer backbone may provide a small increase in the local kinetic energy of the system, which could act to stabilize the liquid phase. As the temperature decreases, the intramolecular motion decreases, until longer-lasting hydrogen bonds start to form between water and hydroxyl groups on the PVA backbone. These hydrogen bonds may “lock” the hydroxyls in place at 0.63 Å farther apart than on the native backbone, which induces a net strain on the MPVA (2) molecule. The combination of supplemental kinetic energy due to polymeric intramolecular motion and the additional energy required to strain the MPVA (2) molecule therefore creates an energy barrier to crystallization that inhibits nucleation. This is especially noticeable when more molecules of MPVA (2) were present, which delayed or possibly inhibited ice nucleation altogether, as shown in Figure 6.

In contrast, when the hydroxyl distance on the PVA backbone is either smaller or larger than that of liquid water, as in MPVA (4), the intramolecular motion of the PVA chain may create a local hydrogen repulsion zone (as hydrogen moves around its oxygen host), which can increase the local potential energy of the system and promote the formation of a lower energy crystalline phase away from the PVA molecule. Once the crystalline network has enveloped an MPVA (4) molecule, the hydroxyl groups are forced to move 0.68 Å closer and are again “locked” into position on the backbone. However, the strain induced in the molecule in this case is negligible due to the increase in the length of the polymer chain.

## 3. Materials and Methods

To observe the effects of PVA on ice nucleation, a series of numerical ice nucleation simulations, with and without PVA molecules, were conducted using Materials Studio molecular dynamics (MD) software. Three different MPVA (10 mer) molecules (MPVA (2–4)) were constructed from a base PVA structure (PVA (1)) using Block Copolymer Builder in Materials Studio, and each MPVA compound was created by varying the distances between the hydroxyl (OH) groups, as shown in Figure 2. Hydroxyl distances were calculated in Materials Studio, by measuring the shortest distance between the oxygen atoms for each hydroxyl group after the structures were optimized using geometry optimization. Initial hydroxyl distances were measured before nucleation simulations were conducted, while final distances were measured upon the conclusion of nucleation. Hydroxyl distances were measured for all adjacent hydroxyl groups in all compounds to account for distance variations between the hydroxyl groups, and the ranges and averages are shown in Table 1. Acetyl groups were added to the MPVA compounds, which were designated as MPVA (2), MPVA (3), and MPVA (4), and these modifications were utilized to determine the effects of additional functional groups, as well as to subdivide the hydroxyl groups.

**Figure 8:**
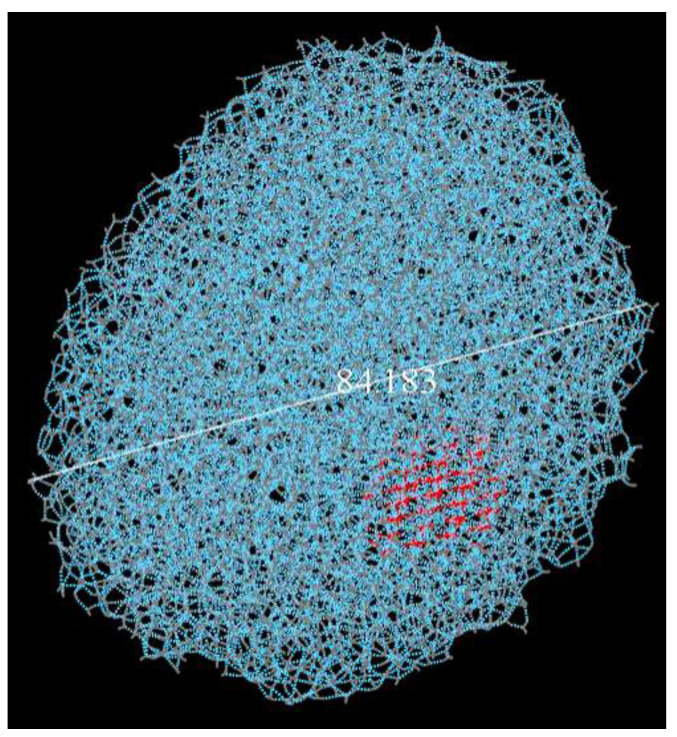
An ~84 Å diameter water droplet with a constrained ice embryo (in red) before molecular dynamics nucleation simulations.

Two sets of simulations were performed for each PVA and MPVA type, with and without a constrained ice embryo present. Molecular dynamics simulations were constructed by manually placing the PVA on a pre-constructed 84.183 Å diameter water droplet, which consisted of 1705 water molecules. The water droplet used in the ice nucleation simulations was constructed by using a pre-constructed ice crystal from the Materials Studio library. A small section of the ice crystal, approximately 16 × 5 × 20 Å in size (Figure 8, in red) was constrained, while the rest was allowed to melt. This constrained section was used as the ice embryo in the constrained simulations (C).

Melting of the ice crystal was accomplished by running Forcite Dynamics NVT (constant number of particles *n*, volume V, and temperature T) at 373 K for a total of 5000 picoseconds (ps), using the COMPASS II forcefield in Materials Studio. Hydrogen bonding distance interactions were set to 19 Å to allow for the surrounding water molecules to coalesce and form a water droplet approximately 84 Å in diameter, as shown in Figure 8.

The ice embryo was initially left unconstrained (Figure 8, in red) and was allowed to melt in the first set of simulations but was then left constrained for the second set as it helped to facilitate nucleation. Parameters for the molecular dynamics simulations were kept constant among all the nucleation simulations for consistency. After initial construction, but before nucleation simulations, all compounds used in this study were optimized for geometry for 5000 ps using the Newton–Raphson method for structure optimization.

All nucleation simulations utilized the COMPASS II forcefield and were run using Forcite Dynamics: the module that controls the classical molecular dynamics simulations in Materials Studio. Bonding distance was set to 19 Å, and all simulations were run at standard atmospheric pressure. These simulations were first run at NVE (constant number of particles *n*, volume V, and energy E) for 5.000 picoseconds (ps), followed by NVT for a total of 15,000 picoseconds (ps). Temperature was held constant at 250 K, with an undercooling of 23 K, and quasi Newton–Raphson was utilized for all Forcite Dynamics nucleation simulations. Molecular dynamics simulations of the water droplet were conducted first to ensure nucleation occurred consistently with just pure water, followed by simulations containing the PVA and MPVA compounds.

Previous studies [28,29,30,31] have evaluated the free energy profile in order to determine the crystallization of a nucleus. Classical nucleation theory (CNT) states that the equation for the Gibbs energy barrier G of a spherical cluster consisting of *n* particles can be expressed by [30]:

∆G(*n*) = −*n*∆fusµ + γ [36π(*n*/ρ)2] 1/3 (1)

where ρ is the number density of the crystalline phase, ∆fusµ is the change in chemical potential on fusion, and γ is the interfacial free energy between the two phases. Thus, an estimation of the applicability of CNT can be obtained for numerical ice nucleation systems [30]. Because changes in the potential energy of the system can be used as an indicator of nucleation, as shown by Matsumoto, et. al., our MD simulations were monitored for the total potential energy of each system before, during, and after nucleation [28]. Final NVT simulation results were thus recorded during both initiation and completion of nucleation, and these were plotted as a function of time (picoseconds), as shown in Figure 3a–j.

While only one PVA or MPVA molecule type was included for the initial nucleation simulations, a final simulation with four molecules of MPVA (2) was conducted following the results of the initial nucleation simulations. This four-molecule model was included because ice nucleation inhibition is directly affected by the concentration of IRI compounds, and the additional nucleation simulation was therefore conducted to account for MPVA compound concentration [18,19,20,21,22,23,24,25].

## 4. Conclusions

The promotion, delay, or even inhibition of ice nucleation was shown to be greatly affected by the presence of ice embryos, as well as PVA and MPVA structure, concentration, and hydroxyl group separation distance. Ice nucleation was initiated sooner when a constrained ice embryo was present in all cases. However, MPVA (2), which had an initial hydroxyl distance of 5045 Å, delayed ice nucleation by a much greater margin compared to other PVA and MPVA molecules. When a greater concentration of MPVA (2) molecules was simulated, ice nucleation did not occur in the simulation timeframe of 15,000 ps, indicating possible nucleation inhibition activity of MPVA (2). However, further investigation will be required to determine the extent of delay in ice nucleation and whether the system reached equilibrium without nucleating.

Therefore, because IRI activity was found to occur when the hydroxyl group distance was between 2858 Å and 7.5117 Å, new anti-freeze compounds could be constructed based upon this example, as long as the underlying backbone composition or overall structure remains flexible.

## Figures and Tables

**Table 1:** Measured average hydroxyl distances for PVA (1), MPVA (2), MPVA (3), and MPVA (4) after nucleation.

Compound	Average Initial Hydroxyl Distance and Range (Å)	Average Final Hydroxyl Distance and Range (Å)
PVA 1	2547 (2524–2631)	2828 (2527–2989)
MPVA 2	5045 (5029–5073)	5725 (5579–5834)
MPVA 3	2667 (2652–2735)	2855 (2780–2026)
MPVA 4	7747 (7596–7899)	7117 (6986–7248)
